# Amyloid PET across the cognitive spectrum in former professional and college American football players: findings from the DIAGNOSE CTE Research Project

**DOI:** 10.1186/s13195-023-01315-5

**Published:** 2023-10-05

**Authors:** Robert A. Stern, Diana Trujillo-Rodriguez, Yorghos Tripodis, Surya V. Pulukuri, Michael L. Alosco, Charles H. Adler, Laura J. Balcer, Charles Bernick, Zachary Baucom, Kenneth L. Marek, Michael D. McClean, Keith A. Johnson, Ann C. McKee, Thor D. Stein, Jesse Mez, Joseph N. Palmisano, Jeffrey L. Cummings, Martha E. Shenton, Eric M. Reiman, for the DIAGNOSE CTE Research Project Investigators, Kewei Chen, Hillary Protas, Yi Su, Connie Boker, Rhoda Au, Robert C. Cantu, Lindsay Farrer, Robert Helm, Douglas I. Katz, Neil Kowall, Gustavo Mercier, James Otis, Jason Weller, Tahlia Bragg, Irene Simkin, Suzan van Amerongen, Alondra Andino, Shannon Conneely, Courtney Diamond, Tessa Fagle, Olivia Haller, Tennyson Hunt, Nicole Gullotti, Bailey Kossow, Carrie Kugelmass, Megan Mariani, Brian Mayville, Kathleen McLaughlin, Mary Nanna, Marty DiPopolo, Taylor Platt, Fiona Rice, Madison Sestak, Douglas Annis, Christine Chaisson, Diane B. Dixon, Carolyn Finney, Kerrin Gallagher, Kaitlin Hartlage, Jun Lu, Brett Martin, Emmanuel Ojo, Brittany Pine, Janani Ramachandran, Fatima Tuz-Zahra, Eukyung Yhang, Sylvain Bouix, Jennifer Fitzsimmons, Alexander P. Lin, Inga K. Koerte, Ofer Pasternak, Hector Arciniega, Tashrif Billah, Elena Bonke, Katherine Breedlove, Holly Carrington, Eduardo Coello, Michael J. Coleman, Omar John, Leonard Jung, Huijun Liao, Maria Loy, Elizabeth Rizzoni, Vivian Schultz, Annelise Silva, Brynn Vessey, Tim L. T. Wiegand, Sarah Banks, Jason Miller, Aaron Ritter, Marwan Sabbagh, Raelynn de la Cruz, Jan Durant, Morgan Golceker, Nicolette Harmon, Jaeson Kaylegian, Rachelle Long, Christin Nance, Priscilla Sandoval, Miranda Staples, Robert W. Turner, Emma F. Clark, Andrew Serrano, David W. Dodick, Yonas Geda, Jennifer V. Wethe, Amy Duffy, Bryce Falk, Marci Howard, Michelle Montague, Thomas Osgood, Debra Babcock, Patrick Bellgowan, William Barr, Judith Goldberg, Binu Joseph, Ivan Kirov, Yvonne Lui, Charles Marmar, Thomas Wisniewski, Alhassan Al-Kharafi, Allan George, Lisena Hasanaj, Sammie Martin, Edward Riley, William Runge, Liliana Serrano, Nicholas Ashton, Henrik Zetterberg, Kaj Blennow, Jeffrey Iliff, Gail Li, Deidre Janssen, James Meabon, Elaine R. Peskind, Juan Piantino, Abigail Schindler, Ronald Thomas, Elizabeth Colasurdo, Jane Shofer, Daniel S. Marcus, Jenny Gurney, Richard Greenwald

**Affiliations:** 1 Boston University CTE Center, Boston University Chobanian & Avedisian School of Medicine, 72 E. Concord Street, Boston, MA L525 USA; 2 Boston University Alzheimer’s Disease Research Center, Boston University Chobanian & Avedisian School of Medicine, Boston, MA USA; 3 Department of Neurology, Boston University Chobanian & Avedisian School of Medicine, Boston, MA USA; 4 Departments of Neurosurgery, and Anatomy & Neurobiology, Boston University Chobanian & Avedisian School of Medicine, Boston, MA USA; 5 Graduate Program in Neuroscience, Boston University Chobanian & Avedisian School of Medicine, Boston, MA USA; 6 Department of Biostatistics, Boston University School of Public Health, Boston, MA USA; 7 Department of Neurology, Mayo Clinic College of Medicine, Mayo Clinic Arizona, Scottsdale, AZ USA; 8 Departments of Neurology, Population Health and Ophthalmology, NYU Grossman School of Medicine, New York, NY USA; 9 Cleveland Clinic Lou Ruvo Center for Brain Health, Las Vegas, NV USA; 10 Institute for Neurodegenerative Disorders, Invicro, LLC, New Haven, CT USA; 11 Department of Environmental Health, Boston University School of Public Health, Boston, MA USA; 12 Massachusetts General Hospital, Harvard Medical School, Gordon Center for Medical Imaging, Brigham and Women’s Hospital, Boston, MA USA; 13 VA Boston Healthcare System, Boston, MA USA; 14 Department of Pathology and Laboratory Medicine, Boston University Chobanian & Avedisian School of Medicine, Boston, MA USA; 15 Framingham Heart Study, Boston University Chobanian & Avedisian School of Medicine, Boston, MA USA; 16 Biostatistics and Epidemiology Data Analytics Center (BEDAC), Boston University School of Public Health, Boston, MA USA; 17 Department of Brain Health, School of Integrated Health Sciences, Chambers-Grundy Center for Transformative Neuroscience, University of Nevada Las Vegas, Las Vegas, NV USA; 18 Psychiatry Neuroimaging Laboratory, Harvard Medical School, Departments of Psychiatry and Radiology, Brigham and Women’s Hospital, Boston, MA USA; 19 Banner Alzheimer’s Institute, University of Arizona, Arizona State University, Translational Genomics Research Institute, and Arizona Alzheimer’s Consortium, Phoenix, AZ USA

**Keywords:** American Football, Amyloid-β, Concussion, Alzheimer’s disease, Cognitive function, Chronic traumatic encephalopathy, Dementia, Florbetapir, Neurodegenerative disease, Positron emission tomography, Repetitive head impacts, Subconcussive trauma, Tau, Traumatic brain injury

## Abstract

**Background:**

Exposure to repetitive head impacts (RHI) in American football players can lead to cognitive impairment and dementia due to neurodegenerative disease, particularly chronic traumatic encephalopathy (CTE). The pathognomonic lesion of CTE consists of perivascular aggregates of hyper-phosphorylated tau in neurons at the depths of cortical sulci. However, it is unclear whether exposure to RHI accelerates amyloid-β (Aβ) plaque formation and increases the risk for Alzheimer’s disease (AD). Although the Aβ neuritic plaques characteristic of AD are observed in a minority of later-stage CTE cases, diffuse plaques are more common. This study examined whether former professional and college American football players, including those with cognitive impairment and dementia, have elevated neuritic Aβ plaque density, as measured by florbetapir PET. Regardless of cognitive and functional status, elevated levels of florbetapir uptake were not expected.

**Methods:**

We examined 237 men ages 45–74, including 119 former professional (PRO) and 60 former college (COL) football players, with and without cognitive impairment and dementia, and 58 same-age men without a history of contact sports or TBI (unexposed; UE) and who denied cognitive or behavioral symptoms at telephone screening. Former players were categorized into four diagnostic groups: normal cognition, subjective memory impairment, mild cognitive impairment, and dementia. Positive florbetapir PET was defined by cortical-cerebellar average SUVR of ≥ 1.10. Multivariable linear regression and analysis of covariance (ANCOVA) compared florbetapir average SUVR across diagnostic and exposure groups. Multivariable logistic regression compared florbetapir positivity. Race, education, age, and APOE4 were covariates.

**Results:**

There were no diagnostic group differences either in florbetapir average SUVR or the proportion of elevated florbetapir uptake. Average SUVR means also did not differ between exposure groups: PRO-COL (*p* = 0.94, 95% C.I. = [− 0.033, 0.025]), PRO-UE (*p* = 0.40, 95% C.I. = [− 0.010, 0.029]), COL-UE (*p* = 0.36, 95% CI = [0.0004, 0.039]). Florbetapir was not significantly associated with years of football exposure, cognition, or daily functioning.

**Conclusions:**

Cognitive impairment in former American football players is not associated with PET imaging of neuritic Aβ plaque deposition. These findings are inconsistent with a neuropathological diagnosis of AD in individuals with substantial RHI exposure and have both clinical and medico-legal implications.

**Trial registration:**

NCT02798185.

**Supplementary Information:**

The online version contains supplementary material available at 10.1186/s13195-023-01315-5.

## Background

The routine play of American football involves exposure to repetitive head impacts (RHI), including those resulting in symptomatic concussions and the more common asymptomatic subconcussive trauma [1–3]. This RHI exposure can lead to cognitive impairment and dementia later in life [4–8], and to the neurodegenerative disease, chronic traumatic encephalopathy [9–13] (CTE). CTE is currently diagnosed through postmortem neuropathological evaluation, characterized by the perivascular deposition of hyper-phosphorylated tau (p-tau) in neurons (with or without astrocytic involvement) at the depths of the cortical sulci, with widespread p-tau deposition and neurodegeneration in later stages [9, 14–17]. CTE is a unique disease that is distinct from Alzheimer’s disease (AD) and other tauopathies; this distinction is based on the type and distribution of p-tau deposition [14, 18], molecular structure of the tau filament [19, 20], specific location and evolution of p-tau isoforms [21, 22], and tau phosphorylation sites [23].

Similar to AD and other neurodegenerative diseases [24–26], CTE often has concomitant neurodegenerative and aging-related pathologies [27–32]. Although amyloid-β (Aβ) diffuse plaques are present in 52% of post-mortem CTE cases [33], moderate-to-frequent Aβ neuritic plaques, a defining characteristic of AD, are found in only 14% of confirmed cases of CTE, predominantly in older individuals with later stage pathology [15]. Results of in vivo amyloid positron emission tomography (PET) have been reported in two small studies of cognitively impaired former football and other contact sport athletes and have not found significantly elevated tracer binding compared to asymptomatic controls [34, 35]. These preliminary studies had several limitations (e.g., small sample size, inconsistent sources and levels of RHI exposure, limited range of cognitive impairment), however, precluding conclusions as to whether cognitive impairment and dementia in former American football players are associated with Aβ neuritic plaque pathology.

Here, we report findings from the Diagnostics, Imaging, and Genetics Network for the Objective Study and Evaluation of Chronic Traumatic Encephalopathy (DIAGNOSE CTE) Research Project, a multi-center, 8-year study funded by the National Institute of Neurological Disorders and Stroke (NINDS). A detailed description of the study design, procedures, and sample (including detailed inclusion and exclusion criteria) has been previously reported [36]. The aim of the cross-sectional study reported herein was to assess neuritic amyloid plaque density, as measured by florbetapir PET, in former professional and college football players across the spectrum of cognitive functioning (unimpaired, subjective memory complaints, mild cognitive impairment [MCI], and dementia), and in same-age asymptomatic men without exposure to RHI. Based on results of previous small studies [34, 35], and because deposition of neuritic plaques is not a common or early neuropathological finding in American football players [15, 33]—even in those with memory impairment and mild dementia—we hypothesized that florbetapir uptake would *not* be elevated in the former players compared to unexposed controls and that florbetapir uptake would not be associated with length of RHI exposure or to cognitive impairment or dementia.

## Methods

### Sample

The DIAGNOSE CTE Research Project includes 120 former professional American football players (PRO), 60 former college football players (COL), and 60 same-age men without a history of playing football or other RHI exposure (i.e., unexposed [UE]). All participants were male and 45–74 years old. Three participants did not receive florbetapir PET due to dose failure at the manufacturer. Therefore, the sample for this study consisted of 237 men, including 119 PRO, 60 COL, and 58 asymptomatic UE. PRO participants played a minimum of 12 years of organized football, including ≥ 3 in college and > 3 seasons in the National Football League (NFL). COL participants played organized football for ≥ 6 years, with  ≥3 years at the college level, and had no contact/collision sports involvement following college. UE participants had no history of participation in contact/collision sports or combat military service. At telephone screening, all UE participants denied cognitive, mood, or behavioral symptoms; functional dependence; history of concussion or traumatic brain injury (TBI); or preexisting psychiatric disorders. Data were collected between September 2016 and February 2020. All participants provided written informed consent.

### Diagnostic classification

Specific tests, variables, and cutoff scores for this study were determined a priori to group participants into one of four categories: cognitively normal (CN), subjective memory complaints (SMC), mild cognitive impairment (MCI), and dementia (DEM) across the cognitive continuum [37]. The algorithm used for this categorization is detailed in the Supplementary Material (Additional File 1). Participants who did not fall within any of the four diagnoses (*n* = 18) were excluded from analyses involving diagnostic grouping but included in other analyses. The Test of Memory Malingering (TOMM) [38] was used to detect suboptimal effort on neurocognitive tests. For all analyses other than exposure group differences (i.e., PRO, COL, UE), participants with a TOMM trial 2 score ≤ 45 (*n* = 11, 1 missing) were excluded.

### Florbetapir PET acquisition and evaluation

Florbetapir doses at the four DIAGNOSE performance evaluation sites were requested through and provided at no cost by Avid Radiopharmaceuticals (Philadelphia, PA, USA). Quality control and imaging calibration procedures were completed prior to study initiation by Invicro (Needham, MA, USA). PET imaging involved a 10-min acquisition (10 frames, 1 min in length each) following a 370 MBq (10 mCi) bolus injection of florbetapir. Fifty minutes after injection, a second 15-min scan was done (acquired in 3 × 5-min frames). Images were uploaded to Invicro for quality control checks. Cortical-cerebellar florbetapir standardized uptake value ratios (SUVR) were calculated as published elsewhere [39, 40]. A positive florbetapir PET scan was defined by an average cortical SUVR of 1.10 or greater (centiloid values > 24.3), equivalent to moderate-to-frequent neuritic Aβ plaques [41]. A negative PET scan was defined by an SUVR < 1.10, indicating sparse-to-no neuritic amyloid plaques.

### Statistical analysis

Power calculations, based on reports of using florbetapir to assess amyloid burden in AD dementia, MCI, and normal aging [42], indicate that 35 participants per group would result in 80% power to detect a significant group difference in average SUVR of at least 0.15 (MCI and CN SUVR = 1.2 vs 1.05). For exposure analyses, the PRO and COL groups were combined. Multivariable linear regressions were conducted for comparison of florbetapir SUVR and its association with exposure. Multivariable logistic regressions were conducted for group comparisons of florbetapir positivity. For bivariate analyses, *t*-tests were used for florbetapir SUVR and chi-square tests for florbetapir positivity. The following variables were controlled for in all models: age, race, years of education, and APOE ε4 genotype (carrier vs. non-carrier). Statistical significance was set at a more liberal *P* < 0.05 to reduce type 2 error because of the hypothesized nonsignificant results. Statistical analyses were conducted using R version 4.00.

## Results

**Fig. 1:**
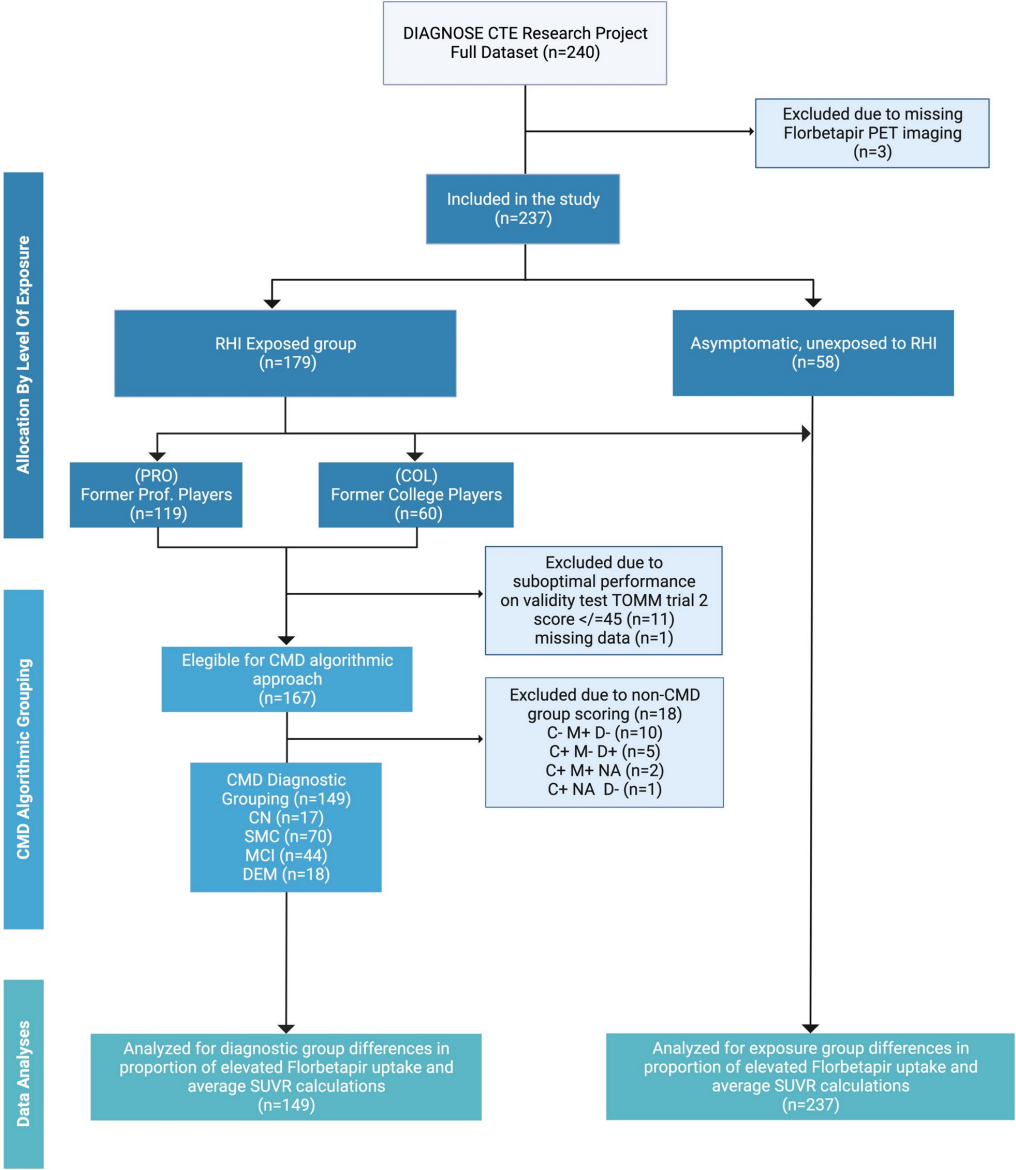
Study flow chart illustrating participant recruitment and allocation to repetitive head impact exposure groups and diagnostic groups for data analyses. Abbreviations: RHI, repetitive head impacts; CMD, Complaints-Memory-Dementia algorithmic grouping; CN, cognitively normal; SMC, subjective memory complaint; MCI, mild cognitive impairment; DEM, dementia; SUVR, standard uptake value ratio; NA, not applicable; TOMM, Test of Memory Malingering, CMD algorithmic approach for diagnostic grouping: Presence or absence of subjective cognitive complaints, C ± (C + defined by Cognitive Complaint Index [memory items] self-report score > 16); presence or absence of objective memory impairment, M ± (M + defined by NAB List Learning Test Delayed Recall T score ≤ 35); presence or absence of dependence in daily functioning, D ± (D + defined as Functional Activities Questionnaire score ≥ 9)

A flow diagram depicting the sampling of subjects, diagnostic status, and inclusion in data analyses is provided in Fig. 1. Participant characteristics by exposure group, including clinical ratings and cognitive test scores, APOE ε4 genotype, and florbetapir average SUVR and percent positive, are summarized in Table 1. There were no significant differences between the exposure groups in the proportion of positive amyloid PET scans or in the continuous florbetapir average SUVR by age range or exposure group (Supplementary Table 1, Additional File 1). Post hoc analysis comparing pairs of exposure groups found that the mean value of florbetapir average SUVR did not differ between PRO and COL (− 0.01 *p* = 0.95, 95% CI [− 0.033, 0.025]), PRO and UE (0.02 *p* = 0.41, 95% CI [− 0.010, 0.029]), and COL and UE (0.02 *p* = 0.36, 95% CI [0.0004, 0.039]) (Table 2). There was no significant association between florbetapir average SUVR and total years of football or with measures of cognition and daily functioning (Supplementary Table 2, Additional File 1).

**Table 1:** Demographics and descriptive data

Characteristic	Former professional football players (PRO) (*n* = 119)	Former college football players (COL) (*n* = 60)	Unexposed (UE) (*n* = 58)	*P* value
Age, mean (SD), years	59.0 (7.8)	53.5 (7.7)	59.5 (8.3)	< 0.001^a^
Age by decade, *n* (%) within exposure group				
45–54	42 (35.3)	44 (73.3)	22 (37.9)	< 0.01
55–64	46 (38.7)	9 (15.0)	14 (24.1)	
65–74	31 (26.1)	7 (11.7)	22 (37.9)	
Body Mass Index, mean (SD) kg/m^2^	32.0 (4.5)	33.8 (4.8)	30.7 (4.4)	0.001^b^
Education, mean (SD), years	16.6 (1.1)	17.1 (1.9)	17.3 (3.5)	0.07
Racial Identity, No. (%)				
American Indian or Alaska Native	0 (0.0%)	0 (0.0%)	0 (0.0%)	0.003
Black or African-American	52 (43.7%)	11 (18.3%)	24 (41.5%)	
Native Hawaiian or Other Pacific Islander	0 (0.0%)	0 (0.0%)	1 (1.7%)	
White	65 (54.6%)	48 (80.0%)	33 (56.9)	
Multiracial	2 (1.7%)	1 (1.7%)	0 (0.0%)	
Ethnicity, No. (%)				
Hispanic or Latino	3 (2.5%)	0 (0.0%)	0 (0.0%)	0.433
Not Hispanic or Latino	116 (97.5%)	60 (100.0%)	58 (100%)	
Total years of football, mean (SD), years	18.0 (3.4)	11.5 (2.5)	NA	< 0.001
Years of NFL participation, mean (SD), years	7.4 (2.7)	NA	NA	NA
Age of first exposure to football, mean (SD), years	11.6 (2.8)	10.2 (2.6)	NA	0.002
Yrs between end of football play and baseline, mean (SD), years	28.8 (8.5)	31.5 (8.1)	NA	0.043
MoCA total, mean (SD) T score	39.3 (13.3)	42.0 (12.5)	49.0 (9.9)	< 0.001^c^
Cognitive Complaint Index, mean (SD) raw score	32.8 (13.0)	31.4 (12.8)	13.7 (2.2)	< 0.001^d^
Cognitive Complaint Index above cut (> 16), No. (%)	100 (84.0%)	52 (86.7%)	7 (12.1%)	< 0.001
NAB List Learning Test Delayed Recall, mean (SD) T score	36.1 (11.7)	41.38 (13.7)	45.6 (12.5)	< 0.001^e^
NAB List Learning Delayed Recall T score impaired (≤ 35), No. (%)	63 (53.9%)	21 (35.0%)	13 (22.4%)	< 0.001
Functional Activities Questionnaire -Informant, mean (SD) total score	3.9 (5.7)	2.97 (5.0)	0.18 (0.5)	< 0.001^d^
ApoE ε4 genotype carriers, No. (%)^f^	33 (28.6%)	20 (33.9%)	11 (20.4%)	0.27
Florbetapir average SUVR ≥ 1.10, No. (%)	10 (8.4%)	7 (11.7%)	3 (5.2%)	0.44
Florbetapir mean (SD) average SUVR	1.00 (0.09)	0.99 (0.10)	0.98 (0.09)	0.30

Continuous variables compared with *T*-test or analysis of variance (for normally distributed data) or Mann–Whitney *U* or Kruskal–Wallis tests (for non-normally distributed data). Significant analysis of variance post-hoc pairwise group comparisons examined with Student–Newman–Keuls test. Categorical variables compared with chi-square or Fischer’s exact tests

*Abbreviations: MoCA* Montreal Cognitive Assessment, *NAB* Neuropsychological Assessment Battery, *SUVR* standardized uptake value ratio, *NA* not applicable

^a^PRO = UE > COL

^b^PRO = UE < COL

^c^PRO = COL < UE

^d^PRO = COL > UE

^e^PRO < COL < UE

^f^ApoE genotyping unavailable for 10 participants (5 PRO, 1 COL, 4 UE)

**Table 2:** Comparisons of florbetapir average SUVR between exposure groups

Comparison by level of exposure	Estimate (95% CI)	Std. error	*t* value^a^	*P-*value
PRO-COL	− 0.01 (− 0.033, 0.025)	0.02	− 0.31	0.95
PRO-UE	0.02 (− 0.010, 0.029)	0.02	1.28	0.41
COL-UE	0.02 (0.0004, 0.039)	0.02	1.36	0.36

*Abbreviations: PRO* former professional football players, *COL* former college football players, *UE* unexposed control group

^a^One-way ANOVA, post hoc Tukey analysis based on the studentized range distribution. *P*-values were adjusted, controlling for age, education, race, and APOEe4 status

**Fig. 2:**
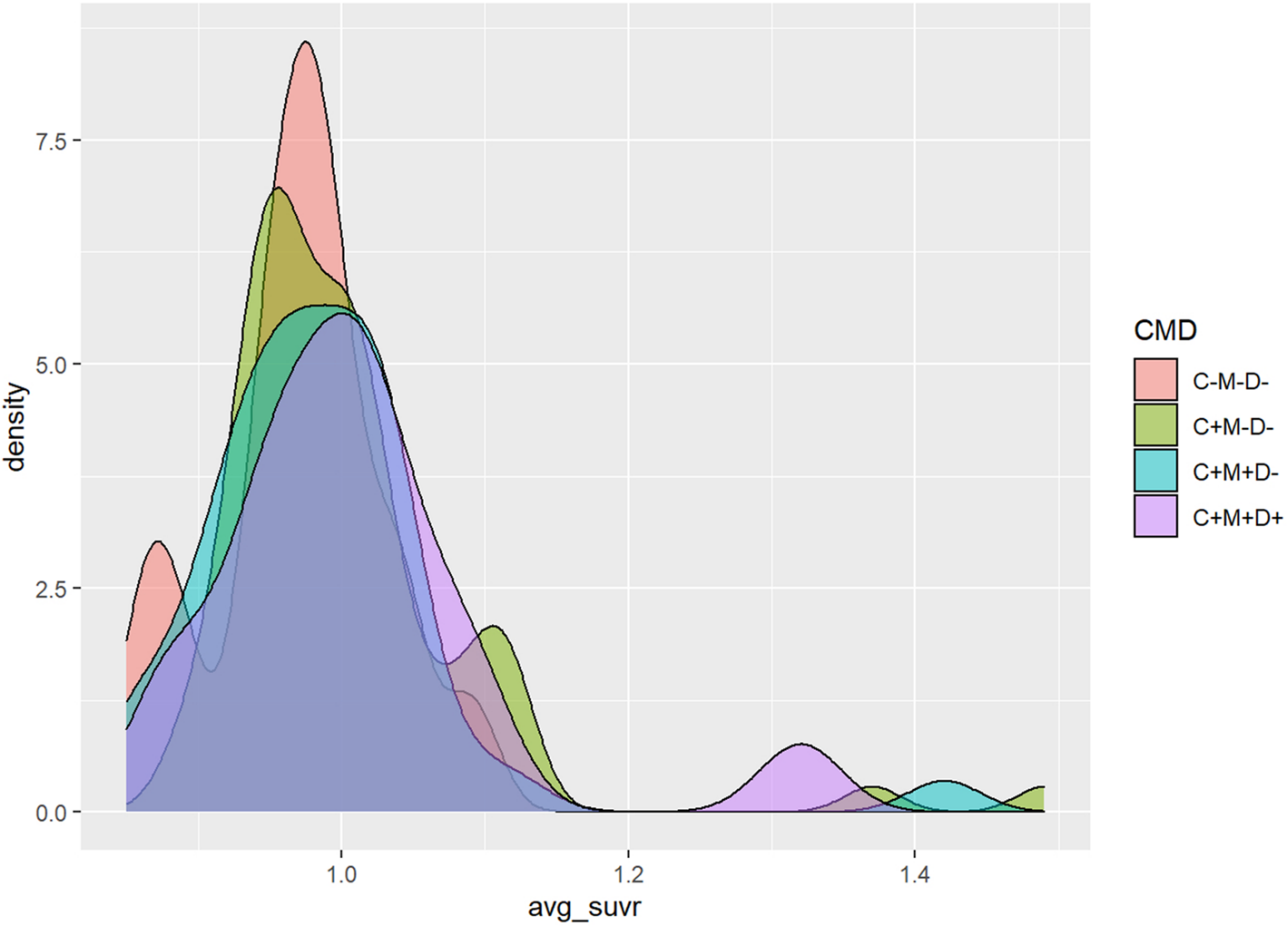
Density map of the mean florbetapir PET average SUVR in former American football players by diagnostic group. Diagnostic groups: C-M-D-, cognitively normal (CN); C + M-D-, subjective memory complaint (SMC); C + M + D-, mild cognitive impairment (MCI); C + M + D + , dementia (DEM). *Y* axis = number of subjects. *X* axis = average SUVR scores. Elevated florbetapir is defined by an average SUVR of 1.10 or greater

Among all former football players (with adequate TOMM performance), there were 17 CN, 70 SMC, 44 MCI, and 18 DEM. None of the CN group had an elevated florbetapir SUVR. There were 9 SMC (13%), 2 MCI (5%), and 2 DEM (11%) with elevated florbetapir SUVRs. Both participants in the DEM group with positive florbetapir scans were former college players though they differed in age and the number of years they played football. One was in the age range of 45 to 54 years and played for over a decade. The other was between 65 to 74 years old but played for less than one decade. Conversely, 16 of the 18 former football players in the DEM group did *not* have elevated florbetapir. Five of them were in the COL group and 11 in the PRO, across the age spectrum. Specific ages of participants are not reported to protect confidentiality. The smoothed density plot in Fig. 2 shows the scaled frequency distribution of florbetapir SUVR in each diagnostic group.

A one-way ANCOVA was conducted to estimate diagnostic group differences among football players (*n* = 149) on the continuous florbetapir average SUVR variable and found no significant between-group differences. Tukey post hoc comparisons found a global *F*-value of 0.94, *p* = 0.43 (Table 3).

**Table 3:** Comparisons of differences in florbetapir average SUVRs between pairs of diagnostic groups in all former football players

Group comparison	Coefficient	Sigma	*T*	*P*
CN-SMC	0.05	0.03	1.86	0.24
CN-MCI	0.02	0.03	0.82	0.84
CN-DEM	0.05	0.03	1.69	0.33
SMC-MCI	− 0.03	0.02	− 1.50	0.43
SMC-DEM	0.01	0.02	0.25	0.99
MCI-DEM	0.03	0.03	1.27	0.57

One-way ANCOVA was performed to assess differences in the four diagnostic groups after controlling the linear effect of covariates by using regression analysis. *F* = 0.94, *p* = 0.43

*Abbreviations: CN* cognitively normal, *SMC* subjective memory complaints, *MCI* mild cognitive impairment, *DEM* dementia

## Discussion

RHI exposure from American football has been associated with later-life cognitive impairment and dementia [4–8], and results of autopsy studies suggest that repetitive brain trauma causes, in part, the neurodegenerative disease, CTE [12, 43]. However, because there are currently no validated in vivo biomarkers for CTE pathology, and because the clinical presentation of CTE is often similar to that of AD, the differential diagnosis of individuals with substantial RHI exposure and later-life cognitive and functional impairment can be difficult [44]. Moderate-to-frequent Aβ neuritic plaques are scarce in CTE [15]. Florbetapir brain PET imaging is approved by the US Food and Drug Administration for estimating Aβ neuritic plaque density for cognitively impaired adult patients being evaluated for AD and other causes of cognitive decline [45]. A negative florbetapir PET indicates sparse-to-no neuritic plaques and is inconsistent with a neuropathological diagnosis of AD. The use of florbetapir and other amyloid PET imaging in clinical settings results in changes in the clinical management of patients, in general, and in changes in etiologic diagnosis from AD to non-AD in patients with negative scans [46, 47].

In this study, we found that most former professional and college football players, ages 45–74 years, with and without cognitive impairment or dementia, did not have in vivo amyloid PET evidence of AD. There were no exposure group differences nor cognitive impairment group differences in florbetapir average SUVR. Moreover, there were no differences between the three exposure groups (PRO, COL, UE) in the proportion of elevated SUVR. Finally, there were no significant associations between florbetapir average SUVR and total years of football or clinical measures. Our findings are consistent with previous preliminary studies [34, 35] and provide strong support that the cognitive impairment and dementia experienced by many former professional and college American football players are not associated with amyloid deposition typical of AD.

The percentage of former football players with positive amyloid PET in this study, across *all* cognitive impairment groups, was markedly lower than published prevalence estimates [48, 49]. In our CN, SMC, MCI, and DEM groups, the percentage of amyloid PET positivity was 0%, 13%, 5%, and 11%, respectively. In contrast, the most recent prevalence estimates of amyloid PET positivity for 60-year-old men (based on pooled data from 85 Amyloid Biomarker Study cohorts) are as follows: normal cognition = 17.6% (95% CI 13.8–22.3); subjective cognitive decline = 19.6% (95% CI 14.1–26.5); MCI = 39.2% (95% CI 32.5–46.4); and clinical AD dementia = 88.4% (95% CI 84.5–91.5) [48]. It is unclear why these former football players do not have amyloid PET evidence of AD, the most common cause of cognitive impairment and dementia in aging. That is, is there a potential mechanism that would reduce or prevent Aβ neuritic plaques in individuals with extensive RHI exposure? Animal models have shown that there is microglial activation with a sustained inflammatory response during and following repetitive mTBI [50–53], but before p-tau pathology [54]. In a postmortem study of young contact sport athletes, an increased number of activated microglia, positively associated with CTE severity, has been reported [55]. It is possible that such a primed inflammatory response has a differential effect on amyloid and tau aggregate formation, preventing or delaying the formation of Aβ neuritic plaque, but accelerating and promoting p-tau neurofibrillary inclusions. Additional research is needed to examine this and other potential mechanisms.

### Strengths

This study has several advantages. Unlike previous reports, this study had a relatively large sample size and was well-powered to detect statistical differences if they existed. Our sample was not limited to former professional football players, but also included former college football athletes who never played professional football or any other contact sport after college. The sample of football players includes participants across the spectrum of cognitive impairment. Participants in the UE group had no history of playing contact sports, combat military experience, or concussion/TBI. Importantly, the sample of former NFL players includes 44% Black-identified participants, which is representative of the approximately 40% Black former players in this age group who played during the era our sample would have played (1967–1996). Moreover, the UE group had a similar representation of Black-identified participants (42%).

### Limitations

This study also has limitations. Due to a lack of power, it is possible that we are not detecting early focal amyloid deposits [56]. Hence, conceivably, a higher proportion of participants were unaccounted for progressive amyloid pathology. Longitudinal studies of cognitively unaffected participants and elevated amyloid accumulation have indicated that elevation in baseline amyloid level is associated with a risk for cognitive decline, suggesting a preclinical stage of Alzheimer’s disease [57, 58]. Because this is a cross-sectional study, evolving amyloid deposition indicating incipient AD neuropathologic changes cannot be estimated in individuals below the cut-off point. Future prospective cohort studies may benefit from optimal threshold corrections [59]. By design, in addition to no RHI exposure, the UE “control” group had no complaints of cognitive, mood, or behavioral impairment at telephone screening. Although the UE group may be appropriate for some analyses aimed at biomarker development and validation, its inclusion in other types of analyses may not be appropriate because of the inability to disentangle the exposure history from the clinical presentation [36]. Additionally, although histories of mood or sleep disorders could affect cognitive and functional measures, we did not include them in the diagnostic classification algorithm used in this study, nor were they included as covariates. The PRO group had a similar age and race composition as the UE group. However, the COL group was significantly younger and included fewer Black participants than either the PRO or UE groups. Although age and race were both used as covariates in all analyses, there still may have been an impact on the findings due to these differences. Similar to almost all research in this area, this study only involved men who played American football. Future studies should include women and individuals with other sources of RHI exposure (e.g., former soccer and rugby players, combat military veterans, and victims of intimate partner violence). The number of participants who met the criteria for the dementia classification was relatively small. Future studies may benefit from including a larger number of former football players with dementia. Finally, the classification of participants into the MCI and DEM groups is limited by being based solely on an algorithm using cutoff scores on specific tests, and not based on a clinical evaluation or adjudicated through a consensus diagnostic conference.

### Clinical implications

CTE is a neuropathological diagnosis. Although other neurodegenerative diseases, such as AD, are also defined by their neuropathological characteristics, the development of sensitive and specific in vivo neuroimaging and fluid biomarkers over the past two decades has resulted in improved clinical diagnostic accuracy and early detection of underlying neuropathology [60, 61]. The clinical disorder associated with CTE is traumatic encephalopathy syndrome (TES). The NINDS consensus diagnostic criteria for TES include a provisional level of certainty for underlying CTE p-tau pathology [44]. However, biomarkers are not included in those criteria because biomarker development for CTE had not reached sufficient maturity to be included. Until there are sensitive and specific biomarkers for CTE pathophysiology, a clinical “diagnosis by exclusion”—similar to older diagnostic criteria for “Alzheimer’s disease” [62, 63]—may be appropriate in some, but not all, circumstances. For example, if a 65-year-old former NFL player presents with significant and progressive cognitive decline (including episodic memory impairment and executive dysfunction), no neuropsychiatric symptoms, and mild-moderate dementia, meeting all NINDS consensus criteria for TES with a probable level of certainty for CTE pathology [44]; has no evidence for another cause of his clinical presentation other than AD or CTE; and has a negative amyloid PET, it may be appropriate for the clinician to suspect that CTE is the underlying cause of the patient’s dementia. In this case, the negative biomarker for AD neuritic plaque would be used to inform the diagnosis by exclusion, as previously suggested [31].

A history of “head injury” is commonly included in lists of important risk factors for AD. Although earlier studies indicated a possible increased risk for *dementia* from a single moderate-to-severe TBI [64], more recent studies have not found postmortem evidence of AD pathology in individuals with previous TBI [65]. The lack of evidence of AD pathology following TBI has been demonstrated by in vivo amyloid PET studies of older community-dwelling volunteers [66] and cognitively impaired military veterans [67]. Additional studies have indicated that TBI history increases the risk for *non-AD* dementia [68] and is associated with non-AD pathologies at postmortem examination [65, 69, 70]. There is a clinical and pathological distinction between AD pathology and TBI-related neurodegeneration. Thus, patients with delayed cognitive and functional decline following a moderate-to-severe TBI may not have AD as the sole cause of their dementia [71, 72].

### Medico-legal implications

There are potential medico-legal implications of the results of this study, including those associated with the highly publicized NFL “Concussion Settlement” [73]. This class action settlement provides substantially higher monetary compensation to former players with a diagnosis of AD than for players with similar cognitive impairment and dementia but without an AD diagnosis. For example, a 62-year-old former player who receives a DSM-5 diagnosis of Major Neurocognitive Disorder due to probable AD by a settlement-qualified neurologist who does not order a florbetapir PET would be eligible for compensation of $950,000 [73]. However, if the same patient were to have a florbetapir amyloid PET as part of the evaluation and the results were negative (i.e., inconsistent with a neuropathological diagnosis of AD [45]), the neurologist would likely not make the probable AD diagnosis, and therefore, the player would be eligible for compensation of only $290,000. That is, the retired player who was given the less precise diagnosis of probable AD made without the florbetapir PET would receive substantially more compensation. The compensation criteria for the NFL settlement may need to be modified based on new medical/scientific findings, including findings that cognitive impairment and dementia in many former NFL players may be caused by non-AD neuropathology.

## Conclusion

In this study from the DIAGNOSE CTE Research Project, we did not find evidence of elevated Aβ neuritic plaque density as measured by florbetapir PET imaging in former professional and college football players, with and without cognitive and functional impairment. These findings suggest that AD is not the cause of the former players’ cognitive decline and dementia. Additional studies are needed to clarify the extent to which cognitive impairment in former football players and persons with a history of repetitive head impacts is related to other neuropathological changes, including CTE. Until sensitive and specific biomarkers for CTE p-tau pathology are available, a diagnosis of TES dementia “consistent with CTE” could be considered in older individuals with a substantial history of RHI exposure, progressive cognitive decline, and functional impairment who have negative amyloid PET imaging.

### Supplementary Information

**Additional file 1: **Algorithm for Diagnostic Categorization. **Supplementary Table 1.** Proportion of Positive Amyloid PET Scans by Age within Exposure Group. **Supplementary Table 2.** Correlations^a^ of Florbetapir PET Average SUVR with Clinical Measures and Years of Football.

## Data Availability

Data from the DIAGNOSE CTE Research Project will be available to qualified investigators through the Federal Interagency Traumatic Brain Injury Research (FITBIR) Informatics System, through the National Institutes of Health Center for Information Technology: https://fitbir.nih.gov/content/access-data. DIAGNOSE CTE Research Project data, including those reported in this study, will also be available to qualified investigators through a project-specific data-sharing portal. Interested investigators should contact Dr. Robert A. Stern, bobstern@bu.edu.

## Abbreviations

Aβ: Amyloid-β
ANCOVA: Analysis of covariance
AD: Alzheimer’s disease
CI: Confidence intervals
CMD: Complaints-Memory-Dementia algorithmic grouping
COL: Former college football players
CN: Cognitively normal
CTE: Chronic traumatic encephalopathy
DEM: Dementia
DIAGNOSE CTE: Diagnostics, Imaging, and Genetics Network for the Objective Study and Evaluation of Chronic Traumatic Encephalopathy
MCI: Mild cognitive impairment
MoCA: Montreal Cognitive Assessment
NA: Not applicable
NAB: Neuropsychological Assessment Battery
NFL: National Football League
PET: Positron emission tomography
PRO: Former professional football players
RHI: Repetitive head impacts
SD: Standard deviation
SMC: Subjective memory complaints
SUVR: Standardized uptake value ratios
TBI: Traumatic brain injury
TOMM: Test of Memory Malingering
UE: Unexposed
