# Supplementary material for: Amyloid PET across the cognitive spectrum in former professional and college American football players: findings from the DIAGNOSE CTE Research Project
Source: Alzheimers Res Ther. 2023 Oct 5;15:166. doi: 10.1186/s13195-023-01315-5 (PMC10552261; doi:10.1186/s13195-023-01315-5)
Supplement: Supplementary file 1 — Additional file 1: Algorithm for Diagnostic Categorization. Supplementary Table 1. Proportion of Positive Amyloid PET Scans by Age within Exposure Group. Supplementary Table 2. Correlationsa of Florbetapir PET Average SUVR with Clinical Measures and Years of Football. [file 13195_2023_1315_MOESM1_ESM.pdf]

Amyloid PET Across the Cognitive Spectrum in Former Professional and College American Football Players: Findings from the DIAGNOSE CTE Research Project

Robert A. Stern, Ph.D.*, Diana Trujillo-Rodriguez, M.S*, Yorghos Tripodis, Ph.D., Surya V. Pulukuri, B.S., Michael L. Alosco, Ph.D., Charles H. Adler, M.D., Ph.D., Laura J. Balcer, M.D., M.S.C.E., Charles Bernick, M.D., Zachary Baucom, Ph.D., Kenneth L. Marek, M.D., Michael D. McClean, Sc.D., Keith A. Johnson, M.D., Ann C. McKee, M.D., Thor D. Stein, M.D., Ph.D., Jesse Mez, M.D., M.S., Joseph N. Palmisano, M.A., M.P.H., Jeffrey L. Cummings, M.D., ScD., Martha E. Shenton, Ph.D., Eric M. Reiman, M.D., for the DIAGNOSE CTE Research Project Investigators

*Shared first authorship.

Funded by a grant from the National Institute of Neurological Disorders and Stroke of the National Institutes of Health (U01NS093334)

Supplementary Materials

Additional File 1

Table of Contents

Algorithm for Diagnostic Categorization *3*

Supplemental Tables *4*

Supplementary Table 1. Proportion[a] of Positive Amyloid PET Scans by Age within Exposure Group 4

Supplementary Table 2. Correlations[a] of Florbetapir PET Average SUVR with Clinical Measures and Years of Football 5

References for Supplementary Materials *6*

Algorithm for Diagnostic Categorization

Specific tests, variables, and cutoff scores for this study were determined *a priori* to group participants into one of four categories: cognitively normal (CN), subjective memory complaints (SMC), mild cognitive impairment (MCI), and dementia (DEM). The Cognitive Complaint Index (CCI, Items 1-12) (1), Neuropsychological Assessment Battery (NAB) List Learning Test delayed recall T-score (2,3), and the Functional Activities Questionnaire (FAQ) total score (4), are included as measures of subjective memory complaints, objective verbal episodic memory, and functional dependence, respectively. A cutoff score of > 16 on the CCI was used to represent meaningful subjective memory complaints (C+). A NAB T-score ≤ 35 (i.e., performance ≥ 1.5 SD worse than age, sex, and education norms) was used to indicate episodic memory impairment (M+). A cutoff of ≥ 9 on the FAQ was used to indicate functional dependence (D+). The following algorithm was used to classify participants: CN = all three measures not beyond cutoff (C–M–D–); SMC = CCI beyond cutoff and NAB and FAQ scores not beyond cutoff (C+M–D–); MCI = CCI and NAB scores beyond cutoff and FAQ score not beyond cutoff (C+M+D–); DEM = CCI, NAB, and FAQ scores all beyond cutoff (C+M+D+).

Supplemental Tables

Supplementary Table 1. Proportion of Positive Amyloid PET Scans by Age within Exposure Group

Characteristic	Former Professional Football Players (PRO) (n=119)	Former College Football Players (COL) (n=60)	Unexposed (UE) (n=58)	*P* value[a]
Positive florbetapir PET scans by decade, n (%)				
45-54	0 (0.00)	1 (5.0)	0 (0.00)	0.08
55-64	5 (25.0)	0 (0.00)	0 (0.00)	
65-74	5 (25.0)	6 (30.0)	3 (15.0)	

Abbreviations: PRO, former professional American football players; COL, former college football players; UE, men without a history of exposure to repetitive head impacts. [a]Based on Pearson's chi-square test.

Supplementary Table 2. Correlations[a] of Florbetapir PET Average SUVR with Clinical Measures and Years of Football

**Predictor**	**Estimate**	***P*-Value**	***n***	**CI**
FAQ	0.09	0.27	158	(-0.07, 0.25)
MoCA T-score	-0.07	0.41	159	(-0.23, 0.09)
NAB LL Trials 1-3 T-score	-0.06	0.49	159	(-0.23, 0.10)
NAB LL Short Delay T-score	0.04	0.62	159	(-0.12, 0.20)
NAB LL Long Delay T-score	0.01	0.94	159	(-0.15, 0.17)
Total years of Football	-0.10	0.17	159	(-0.27, 0.05)

[a]Estimates following *P*-value adjustment based on partial r-squared values representing the squared semi-partial correlations after adjusting for all other background covariates. Abbreviations: FAQ Functional Activities Questionnaire[4] total score; MoCA, Montreal Cognitive Assessment; NAB, Neuropsychological Assessment Battery[3]
Note: Race, education, age, and APOEe4 status were used as background covariates in all models.

References for Supplementary Materials

1. Rattanabannakit C, Risacher SL, Gao S, et al. The Cognitive change index as a measure of self and informant perception of cognitive decline: relation to neuropsychological tests. *J Alzheimer Dis*. 2016;51(4):1145-55. doi:10.3233/jad-150729

2. Gavett BE, Ozonoff A, Doktor V, et al. Predicting cognitive decline and conversion to Alzheimer's disease in older adults using the NAB List Learning test. *J Int Neuropsychol Soc*. Jul 2010;16(4):651-60. doi:10.1017/s1355617710000421

3. Stern RA, White T. *Neuropsychological Assessment Battery*. Psychological Assessment Resources, Inc.; 2003.

4. Pfeffer RI, Kurosaki TT, Harrah CH, Jr., Chance JM, Filos S. Measurement of functional activities in older adults in the community. *J Gerontol*. May 1982;37(3):323-9.
